# Cell Population Kinetics of Collagen Scaffolds in *Ex Vivo* Oral Wound Repair

**DOI:** 10.1371/journal.pone.0112680

**Published:** 2014-11-14

**Authors:** Hermann Agis, Amy Collins, Andrei D. Taut, Qiming Jin, Laura Kruger, Christoph Görlach, William V. Giannobile

**Affiliations:** 1 Periodontics and Oral Medicine, School of Dentistry, University of Michigan, Ann Arbor, Michigan, United States of America; 2 Department of Conservative Dentistry and Periodontology, Medical University of Vienna, Vienna, Austria; 3 Austrian Cluster for Tissue Regeneration, Vienna, Austria; 4 Department of Cariology, Restorative Sciences, and Endodontics, School of Dentistry, University of Michigan, Ann Arbor, Michigan, United States of America; 5 Geistlich Pharma AG, Wolhusen, Switzerland; 6 Department of Biomedical Engineering, College of Engineering, University of Michigan, Ann Arbor, Michigan, United States of America; Université de Technologie de Compiègne, France

## Abstract

Biodegradable collagen scaffolds are used clinically for oral soft tissue augmentation to support wound healing. This study sought to provide a novel *ex vivo* model for analyzing healing kinetics and gene expression of primary human gingival fibroblasts (hGF) within collagen scaffolds. Sponge type and gel type scaffolds with and without platelet-derived growth factor-BB (PDGF) were assessed in an hGF containing matrix. Morphology was evaluated with scanning electron microscopy, and hGF metabolic activity using MTT. We quantitated the population kinetics within the scaffolds based on cell density and distance from the scaffold border of DiI-labled hGFs over a two-week observation period. Gene expression was evaluated with gene array and qPCR. The sponge type scaffolds showed a porous morphology. Absolute cell number and distance was higher in sponge type scaffolds when compared to gel type scaffolds, in particular during the first week of observation. PDGF incorporated scaffolds increased cell numbers, distance, and formazan formation in the MTT assay. Gene expression dynamics revealed the induction of key genes associated with the generation of oral tissue. DKK1, CYR61, CTGF, TGFBR1 levels were increased and integrin ITGA2 levels were decreased in the sponge type scaffolds compared to the gel type scaffold. The results suggest that this novel model of oral wound healing provides insights into population kinetics and gene expression dynamics of biodegradable scaffolds.

## Introduction

A widely used strategy in oral reconstructive surgical procedures is soft tissue augmentation [1]–[3]. Although autologous grafts such as subepithelial connective tissue grafts are successfully applied to increase soft tissue volume, these techniques are associated with several disadvantages. These include increased patient's morbidity due to a second surgical procedure and limited graft material due to anatomical reasons. To overcome these drawbacks of autologous grafts, several strategies using allogeneic and synthetic devices have been developed [4]–[11].

Collagen-based biomaterials are used clinically for guided tissue regeneration (GTR) and soft tissue augmentation [9], [12]–[14]. Collagen, one of the most abundant protein families in human tissue, is biodegradable and supports angiogenesis which makes it an excellent candidate as a scaffold biomaterial [3], [9], [15], [16]. Although biodegradability is important to prevent a second surgery, uncontrolled degradation, and therefore lack of volume stability, is a limitation. Therefore to extend the structural properties and mechanical integrity, cross-linking of collagen has been developed [9], [13], [16], [17].

Although cross-linking of collagen increases the stability of the scaffolds, induction of foreign body reaction has been described [12], [18]. New cross-linking protocols have led to the development of collagen scaffolds that withstand mechanical forces *in vitro* and show a similar clinical outcome compared to subepithelial connective tissue grafts without the induction of foreign body reactions [19], [20]. Currently we are only beginning to understand the cellular responses induced by different scaffold materials and information is still lacking on the healing kinetics and gene dynamics underlying the oral soft tissue graft consolidation [21].

Oral wound healing during tissue regeneration follows a highly conserved sequence of events. Activated platelets release signaling molecules such as platelet-derived growth factor (PDGF). PDGF triggers mesenchymal repair cells to migrate along the growth factor gradient and populate the defect area [22]–[24]. In oral soft tissue grafting, cell-free collagen scaffolds must become populated by cells to support successful augmentation, as opposed to autologous grafts, which already contain cells [1], [3], [19], [20]. Although much effort has been put into the development of these scaffolds, limited data exists on how these devices modulate the cell biological mechanisms underlying this critical early phase of healing. Two- and 3-D *ex vivo* models can shed light on this process and have revealed how growth factors such as PDGF isoforms can support migration and repopulation [23], [24]. However, 3-D *ex vivo* models of oral wound healing that investigate population of the scaffolds are not well-developed.

The aim of this study was to assess the kinetics and gene expression of cells populating cross-linked porous collagen scaffolds (sponge type scaffolds) in a 3-D *ex vivo* model of oral wound healing. To mimic the defect *in vitro*, sponge type scaffolds were evaluated in an artificial “wound area” and compared to gel type scaffolds. To simulate the chemotactic activity of platelets and show that the cells in the system remain responsive to the growth factor, PDGF-loaded scaffolds were also assessed. We evaluated the population kinetics of the scaffolds based on the cell number and distance from the scaffold border of primary human gingival fibroblasts. The proliferation within the defect was measured based on formazan formation using the MTT assay. Changes in gene expression were assessed with gene array and qPCR.

## Material and Methods

### Cell culture

Primary human gingival fibroblasts (hGFs) were purchased from ScienCell Research Laboratories (Corte Del Cedro, Carlsbad, CA, USA). The cells were cultured in Dulbecco's modified Eagle's medium (DMEM; GIBCO-BRL Life Technologies, Grand Island, NY, USA) supplemented with 10% fetal bovine serum (FBS; HyClone Laboratories, Inc, Logan, UT, USA) and penicillin (100 units/mL; GIBCO-BRL) and streptomycin (100 µg/mL; GIBCO-BRL) in a humidified atmosphere of 5% CO^2^ at 37°C. hGFs at passage 5–7 were used for the experiments. For the population experiments the cells were stained with DiIC12(3) (BD Biosciences, Bedford, MA, USA) before they were trypsinized. For gene expression studies, unstained hGFs were used.

### Collagen scaffolds

Cross-linked porous collagen scaffolds (sponge type scaffolds) were kindly provided by Geistlich Pharma (Wolhusen, Switzerland). The sponge type scaffold is composed of porcine collagen type I and porcine collagen type III, with a 93% vol porosity and a mean pore diameter of 92 µm [25]. Cylindrical scaffolds were prepared by first cutting the sponge type scaffolds into 1 mm thick slices, then punching out 6 mm diameter specimens utilizing a biopsy punch (George Tiemann & Co, Hauppauge, New York). The scaffolds were saturated with DMEM with and without platelet-derived growth factor-BB (PDGF; R&D Systems, Minneapolis, MN, USA) at 250 ng/mL and supplemented with 1% FBS, penicillin (100 units/mL; GIBCO-BRL), and streptomycin (100 µg/mL; GIBCO-BRL) before subjecting the scaffolds to the *ex vivo* testing. Scaffolds were compared to collagen type I gels (gel type scaffolds) which are a well-established matrix for 3D cell culture. Gel type scaffolds are composed of rat tail collagen type I (BD Biosciences, Bedford, MA, USA) at 2.5 mg/ml supplemented with DMEM with 1% FBS, penicillin (100 units/mL; GIBCO-BRL) with and without PDGF (R&D Systems) at a concentration of 250 ng/mL. pH was neutralized with NaOH (Sigma-Aldrich, St. Louis, MO, USA).

### Scanning electron microscopy

The morphology of gel type and sponge type scaffolds was evaluated using the Hitachi TM-1000 Scanning Electron Microscope (Hitachi High-Technologies Europe GmbH, Krefeld, Germany) at the Bernhard Gottlieb University Clinic of Dentistry (Medical University of Vienna, Vienna, Austria). The scaffolds were hydrated in phosphate-buffered saline and dehydrated in ethanol as previously described [26]. Then cross-sections were prepared and images were taken at 15,000 accelerating voltage at 400× and 800× magnification.

### *Ex vivo* wound healing model

**Figure 1:**
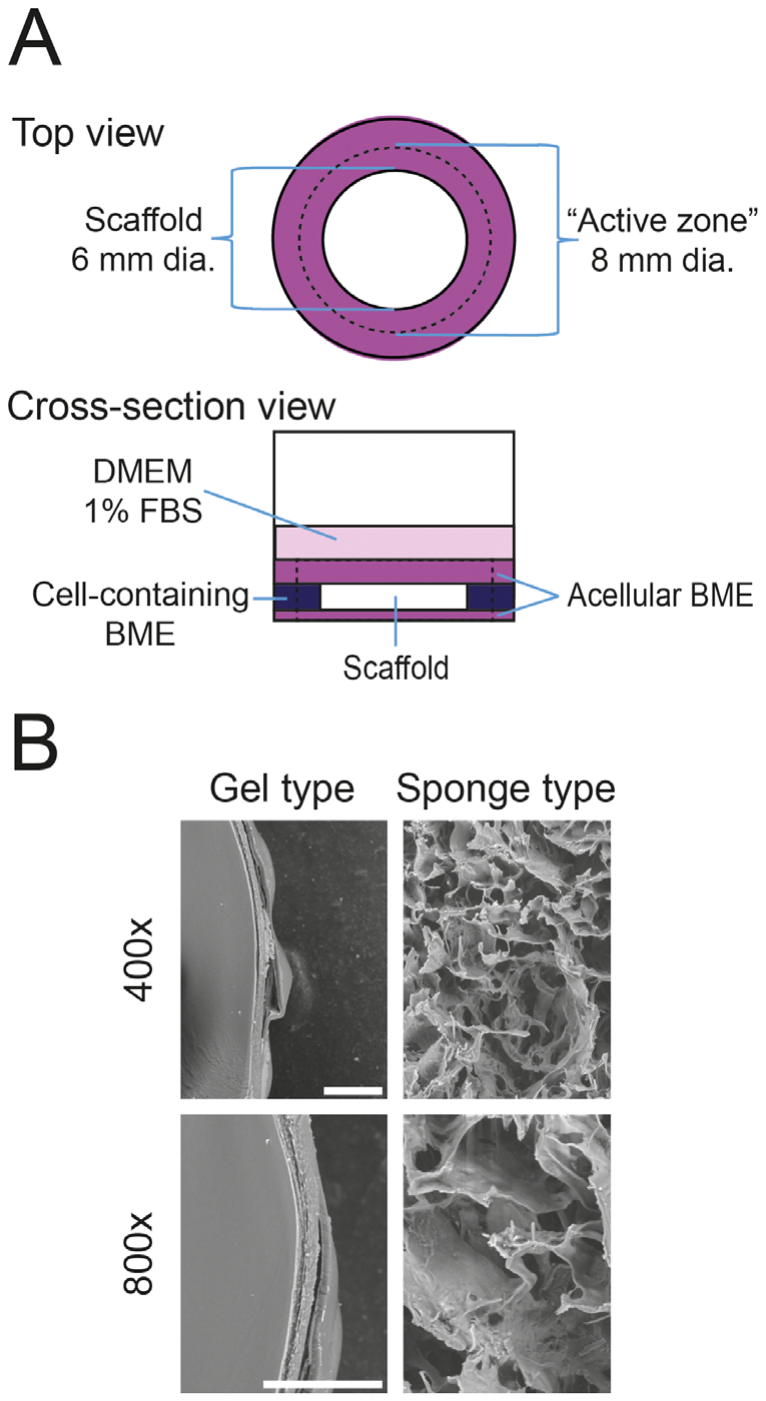
*Ex vivo* wound healing model and scaffold properties. The *ex vivo* wound healing model is shown in top view and cross-sectional view (A). The bottom of the well was covered with an acellular growth factor reduced basal membrane extract (BME). The scaffold (sponge type scaffold composed of cross-linked collagen or the gel type scaffold composed of rat collagen I) in the center was surrounded by a BME-gel containing 800,000 hGFs/ml and covered with an acellular BME gel and Dulbecco's Modified Eagle's Medium (DMEM) supplemented with 1% fetal bovine serum (FBS) and antibiotics. Both scaffold and gel were tested with and without the addition of platelet-derived growth factor-BB (PDGF). Fluorescence images were taken to assess DiI-labeled cells within the scaffold over time. To assess the metabolic activity and gene expression dynamics, the scaffolds including the “active zone” (dashed line) were subjected to MTT tests, gene array analysis, and quantitative PCR. (B) Representative scanning electron microscopy images depict the morphology of the collagen gel, collagen scaffold at 400×, and 800× magnification. The white bar represents 100 µm.

48 well plates were first layered with 50 µL of 12 mg/ml growth factor reduced basal membrane extract (BME; CULTREX, TREVIGEN, Gaithersburg, MD, USA) without cells. For the sponge type scaffolds, the soaked scaffolds were placed in the middle of the well. For the gel type scaffolds, plastic spacers of 6 mm diameter were placed in the middle to generate the defect area. After placement of scaffolds or spacers, the surrounding area was filled with BME supplemented with 800,000 hGF/mL. Following BME polymerization, the plastic spacers were removed and the defect was filled with the collagen gel, with and without 250 ng/ml PDGF and supplemented with DMEM, 1% FBS, and antibiotics. In both groups, the constructs were covered with 150 µL of BME without cells. Finally, after polymerization, the constructs were covered with 200 µL of DMEM supplemented with 1% FBS and antibiotics and incubated in a humidified atmosphere of 5% CO_2_ in air at 37°C for up to 14 days. Figure 1A shows a schematic drawing of the set up as overhead and cross-sectional views.

### Microscopy and image analysis

**Figure 2:**
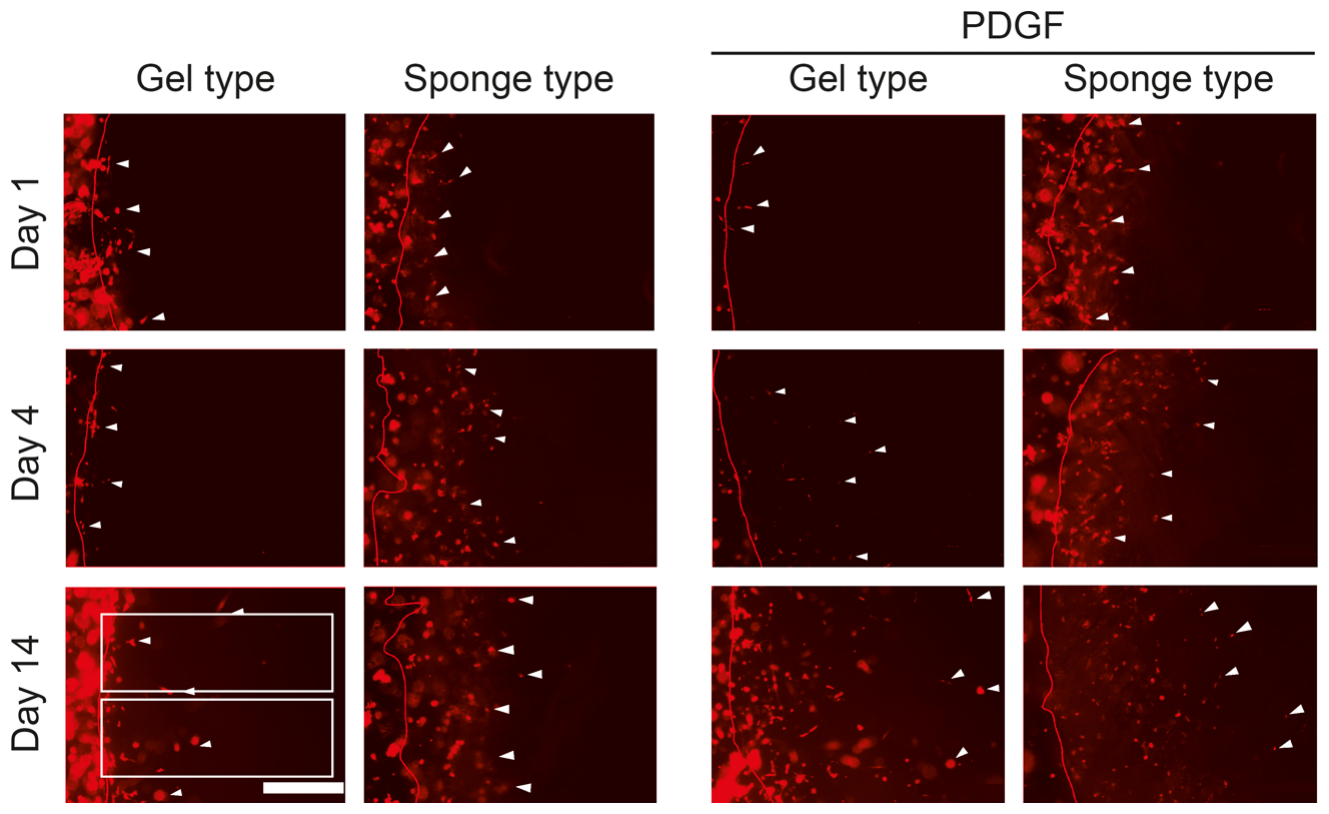
Fluorescence images of human gingival fibroblasts populating the gel type and the sponge type scaffolds. Fluorescence microscopy shows DiI-labeled human gingival fibroblasts populating the gel type and the sponge type scaffolds. Images from days 1, 4, and 14 are shown. White arrows indicate the cells at the migration front. The border of the scaffoldss is indicated by a red line. The white boxes show the regions of interest (0.75 mm^2^), starting at the border of the scaffolds, where quantification of cell numbers and migration distance was performed. The region was 0.5 mm high and 1.5 mm wide. The photos were colorized using Adobe Illustrator. The white bar represents 500 µm.

The DiI-labled hGFs populating the sponge type and gel type scaffolds were examined by bright field and fluorescence microscopy utilizing a Leica DMIRB inverted microscope (Leica Microsystems GmbH, Wetzlar, Germany) at the Microscopy and Image Analysis Laboratory (University of Michigan, Ann Arbor, MI, USA). At days 1, 4, 7, 10, and 14 of culture, photos were taken with an Olympus DP-30 B&W camera (Olympus, Central Valley, PA, USA) using the 5x objective with bright field and fluorescence. The border between BME and scaffolds was identified in the bright field images and transferred onto the fluorescence images using Adobe Illustrator (Adobe Systems, Inc., San Jose, CA USA). Two rectangular regions of interest, 500 µm in width and 1500 µm in length, were defined to measure the cells populating the scaffolds and were positioned perpendicular to the defect margin (Figure 2). The coded images were analyzed by an independent, blinded examiner (AC) using ImageJ (National Institutes of Health, USA). With ImageJ the labeled cells within the regions of interest were counted and their distance to the border of the scaffolds was measured. In total, seven sponge type scaffolds and six gel type scaffolds were evaluated per group. The data are given as mean ± standard deviation.

### Formazan formation

To assess the formazan formation as an indicator of proliferation of the hGFs populating the scaffolds, an MTT assay was used. Briefly, the cells within the *ex vivo* wound healing assembly were exposed to 0.5 mg/mL MTT (3-(4,5-Dimethylthiazol-2-yl)-2,5-diphenyltetrazolium bromide; Sigma, St. Louis, MO, USA) at day 14. After 3 h at 37°C and 5% CO_2_ the samples were washed with PBS and the scaffolds were extracted with an 8 mm diameter punch including the scaffolds and the 1 mm thick “active zone” which surrounded the scaffolds (See Fig 1) and transferred to reaction tubes. By punching out the scaffold and the “active zone” we included the cells in the perimeter of the scaffold. This is in line with the setup of other *ex vivo* wound healing studies [23]. 300 µL isopropanol was added and formazan crystals were solubilized by vortexing for 30 sec. Absorption of 100 µL was measured in a Multiskan Ascent microplate reader (Thermo Labsystems, Waltham, MA, USA) at 560 nm with a 650 nm correction. For the endpoint measurement, eight sponge type scaffolds and seven gel type scaffolds were evaluated. For the time course experiment, six sponge type scaffolds and six gel type scaffolds were evaluated. The data are given as mean ± standard deviation.

### RNA isolation

To assess the gene expression profiles of the cells populating the scaffolds, total RNA was isolated from each sample utilizing the TRIzol method. After briefly washing with PBS, the scaffolds and gel were collected from the *ex vivo* wound healing assemblies at days 1, 4, 7, 10, and 14 with a 8 mm diameter punch to include the scaffold and the surrounding 1 mm thick “active zone” (See figure 1A). In line with the literature, we thereby included the cells in the perimeter of the scaffolds that are in the influence of the scaffolds [23]. Ten samples per group were pooled and transferred into 1 ml TRIzol Reagent (Invitrogen, Carlsbad, CA) for the gene array analysis. Five samples per group were pooled in TRIzol Reagent for the qPCR analysis. Total RNA was extracted following the manufacturer's protocol and quantified by Agilent Technologies Bio-analyzer 2100 (Agilent Technologies, Inc., Santa Clara, CA, USA).

### Gene Array

Gene array analysis was performed using the GeneChip Human Genome U133 Plus 2.0. Array containing over 22,000 probe sets (Affymetrix, Inc., Santa Clara, CA, USA). Total RNA was further purified by RNeasy mini kit (Qiagen, Valencia, CA USA) and measured for quality and quantity by Agilent Technologies Bio-analyzer 2100 (Agilent Technologies, Inc.). 100 ng total RNA per sample was amplified and biotin-labeled according to the manufacturer's instructions. Briefly, RNA was converted to double-stranded complementary DNA (cDNA). Then, cDNA was used in an *in vitro* transcription reaction in the presence of biotin-modified ribonucleotides to produce single stranded RNA (aRNA, Affymetrix, Inc., Santa Clara, CA, USA). The biotin-labeled aRNA was fragmented and 10 µg were hybridized to a GeneChip HumanU133 plus 2.0 (Affymetrix) at 45°C for 16 hours. Labeled bacterial RNAs of known concentration were spiked in the hybridization to generate an internal standard and to allow normalization between chips. Chips were washed, stained and scanned. The data were analyzed by Affymetrix software of Expression Console 1.3 (Affymetrix), and Affymetrix web-based tool of “NetAffx” (Affymetrix) and the gene array data was deposited to the National Center for Biotechnology Information Gene Expression Omnibus database with the access number GSE61314.

### Quantitative polymerase chain reaction

500 ng of total RNA was reverse transcribed into cDNA using SuperScript II Reverse Transcriptase (Invitrogen). cDNA products were amplified using TaqMan PCR Master Mix (Applied Biosystems, Carlsbad, CA) using primers and probes ordered from Applied Biosystems. Reactions were cycled in an ABI PRISM 7700 Sequence Detection System according to the manufacturer. Based on the results of the microarray on day 1 samples, the following genes 25 were evaluated for days 1, 4, 7, 10, and 14: CCL2, CCND1, COL1A1, COL3A1, CTGF, CYR61, DKK1, FN1, HAS2, IGF2, IL-11, ITGA2, ITGB1, MMP1, MMP2, MMP3, MMP14, PDGFRA, PDGFRB, POSTN, TGFBR1, TIMP1, TIMP2, TIMP3, TNC. The full-length gene names, and the specific TaqMan assays are shown in Table S1. Quantification of the data was performed by calculating expression levels of each gene relative to glyceraldehyde 3-phosphate dehydrogenase (GAPDH, HS02758991_g1, Life Technologies) by using the comparative cycle threshold method.

### Statistical analysis

Data are presented as mean ± standard deviation and was analyzed using SPSS using Analysis of Variance (ANOVA) and Fisher's protected least significant difference post hoc test. For the *ex vivo* wound healing assay, the area under the curve (AUC) was calculated for cell numbers and the distance from the scaffold border.

## Results

### Morphology of the scaffolds

Cross-sectional scanning electronic microscopy images at 400-fold and 800-fold magnification revealed a uniform porous structure of the cross-linked sponge type scaffolds (Figure 1 B). In contrast to the sponge type scaffolds, the gel type scaffolds composed of native collagen type I gels contracted dramatically during processing for scanning electronic microscopy and displayed a highly compressed structure in the scanning electronic microscopy images. The difference in stability might be due to the cross-linking of the collagen in the sponge type scaffold. In the *ex vivo* oral wound-healing model, both the gel type scaffolds and the sponge type scaffolds were visible during the entire observation period.

### Cell population kinetics within the scaffolds

**Figure 3:**
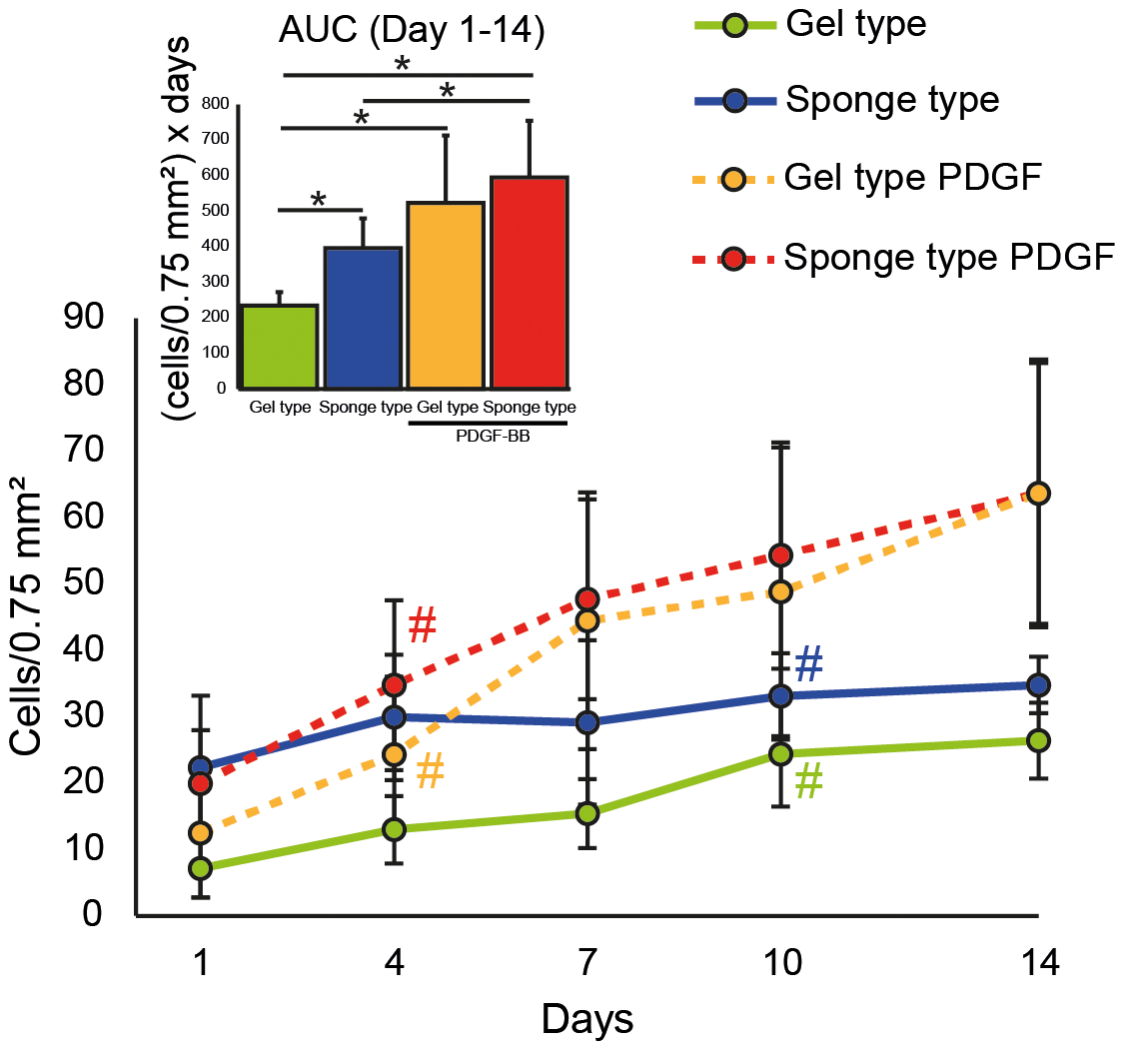
Cell numbers of human gingival fibroblasts in the gel type and the sponge type scaffolds. The number of human gingival fibroblasts in gel type and sponge type scaffolds with and without platelet-derived growth factor-BB (PDGF) was quantified and is presented as mean number of cells ± standard deviation. Area under the curve (AUC) analysis was performed from 1 to 14 days (n = 6–7 per group). Data were analyzed using analysis of variance (ANOVA) and Fisher's protected least significant difference (PLSD) *post hoc* test. Significance (*, #) was assigned at p<0.05. The colored pound sign (#) indicates the first time a difference to day 1 was observed for the group of the corresponding color. The PDGF containing sponge type scaffold was different from the sponge type scaffold from day 7 to day 14. The PDGF containing gel type scaffold was different from the gel type scaffold from day 4 to day 14.

Fluorescence microscopy showed DiI-labeled human gingival fibroblasts, coming from the “active zone” and populating sponge type and gel type scaffolds within one day and increasing throughout the 14-day observation period (Figure 2). Quantification of the cells within the regions of interest revealed that the number of cells in the sponge type scaffolds was higher when compared to the gel type scaffolds at day 1 (p<0.05; Figure 3, see also Table S2 for the corresponding data). This increase remained statistically significant through day 7 (p<0.01). Area under the curve (AUC) analysis revealed an overall increase (p<0.05) of the cell population within the sponge type scaffolds. Addition of PDGF significantly increased the cell number and AUC in both the sponge type and gel type scaffolds (p<0.01).

**Figure 4:**
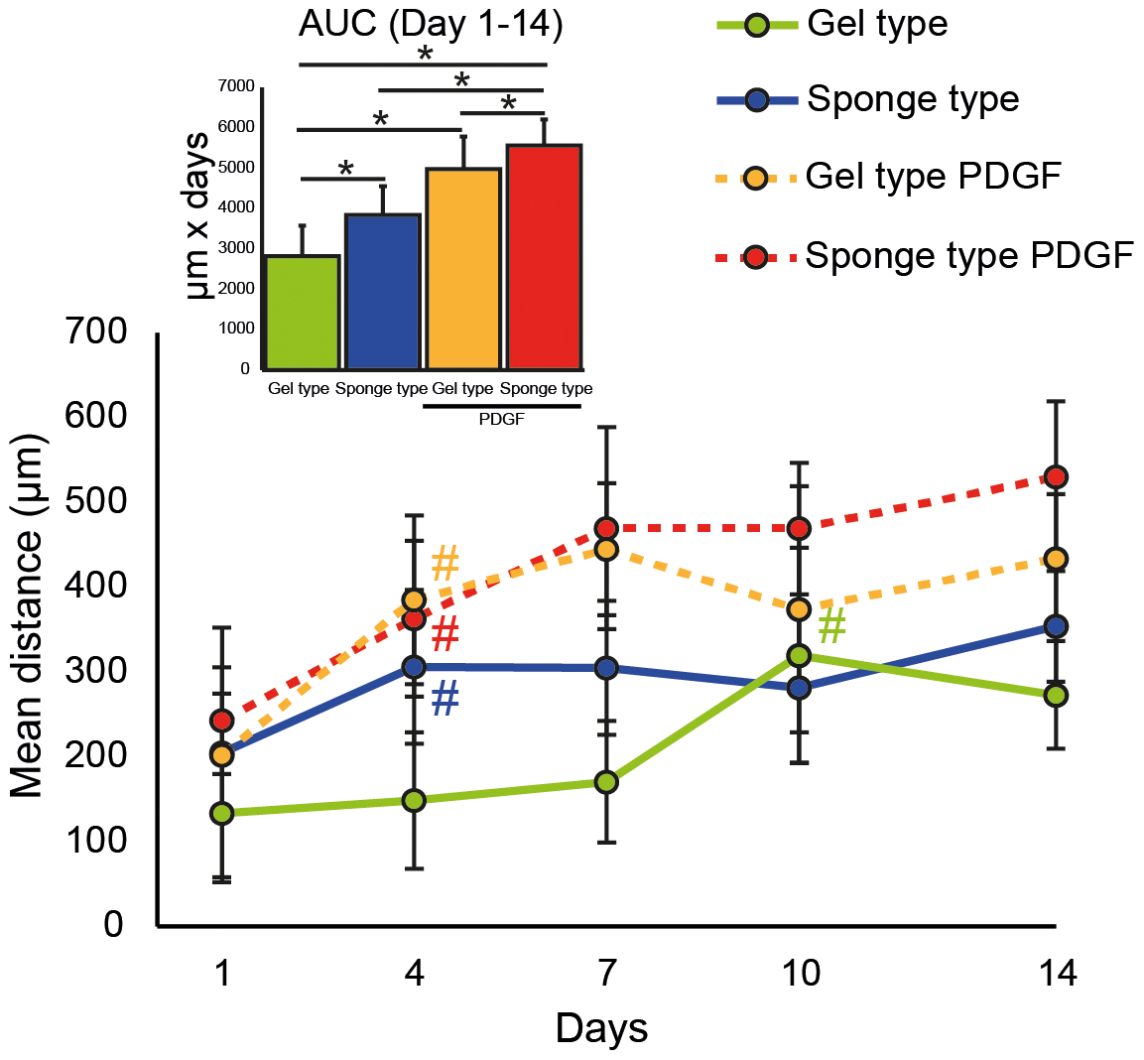
Cell migration distance of human gingival fibroblasts in the gel type and sponge type scaffolds. The migration distance of human gingival fibroblasts in gel type and sponge type scaffolds with and without platelet-derived growth factor-BB (PDGF) was measured and is presented as the mean migration distance from the scaffold border ± standard deviation in the region of interest that was 0.5 mm high and 1.5 mm wide. Area under the curve (AUC) analysis was performed from 1 to 14 days. (n = 6–7). Data was analyzed using analysis of variance (ANOVA) and Fisher's protected least significant difference (PLSD) post hoc test. Significance (*, #) was assigned at p<0.05. Colored pound sign (#) indicates the first time a difference to day 1 was observed for the group of the corresponding color. The PDGF containing sponge type scaffolds was different from the scaffolds alone from day 7 to day 14. The PDGF containing gel type scaffold was different from the gel type scaffold from day 4 to day 14.

Evaluation of the distance from the scaffold border revealed the mean distance of the hGF from the border of the sponge type scaffold within the region of interest increased over time (Figure 4, see also Table S3 for the corresponding data). The mean distance of the hGF within the sponge type scaffolds was greater when compared to the gel type scaffolds at days 4 and 7 (p<0.01). AUC analysis revealed an overall increase (p<0.05) of the mean distance within the scaffolds compared to the gel type scaffolds. Addition of PDGF increased the distance within the sponge type scaffolds from day 7 to day 14 and within the gel type scaffolds from day 4 to day 14 when compared to the corresponding group without PDGF (p<0.01). This increase was also observed when an AUC analysis was performed (p<0.01). AUC analysis further revealed that the distance was greater in the sponge type scaffolds than in the gel type scaffolds (p<0.05).

### Formazan formation

**Figure 5:**
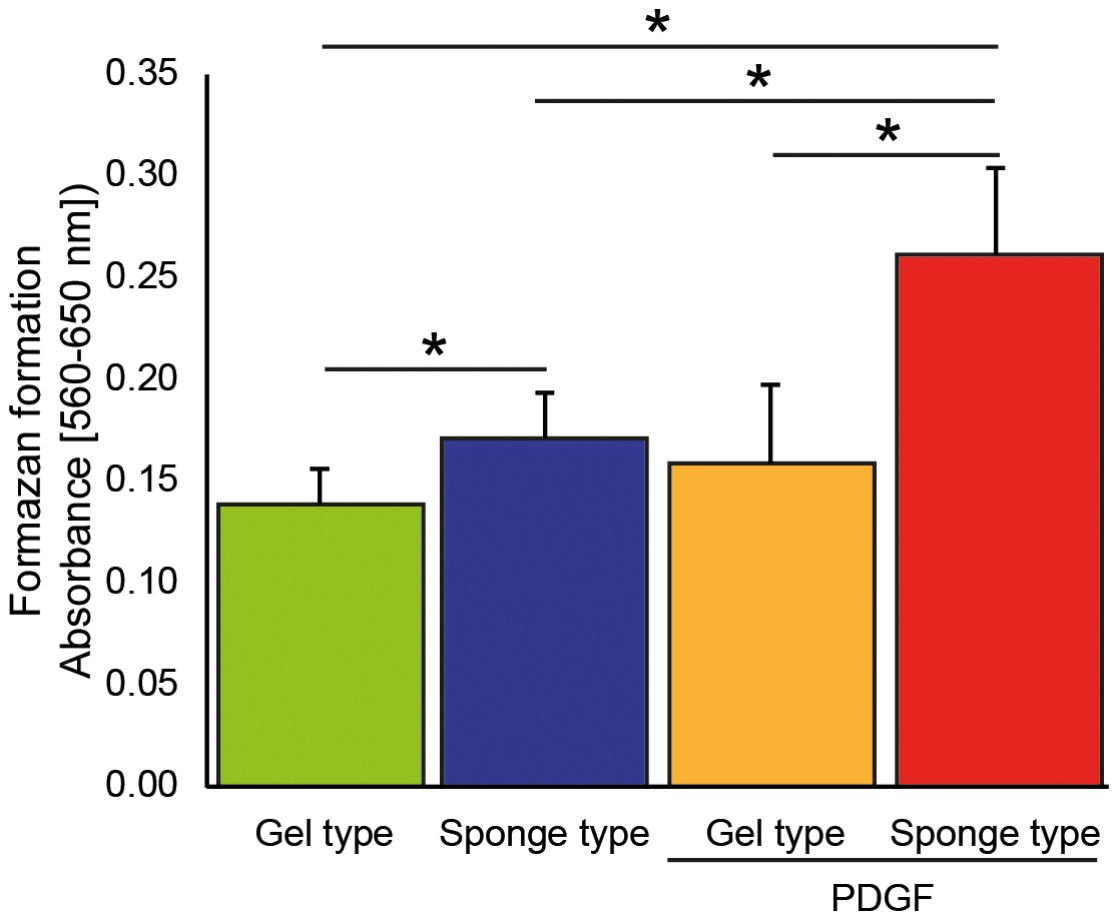
Formazan formation of cells populating the gel type and sponge type scaffolds. Formazan formation in the gel type and sponge type scaffolds with and without platelet-derived growth factor-BB (PDGF) at day 14 was quantified utilizing the MTT assay. Data are given as mean ± standard deviation. (n = 7–8) Data was analyzed using analysis of variance (ANOVA) and Fisher's protected least significant difference (PLSD) *post hoc* test. Significance (*) was assigned at p<0.05.

Proliferation of the hGFs within the scaffolds and the surrounding “active zone” (See figure 1A for a schematic drawing) at day 14 was quantified utilizing the MTT assay. We measured formazan formation as an indicator of proliferation in all 4 groups using the well-established MTT assay [25] (Figure 5, see also Table S4 for the corresponding data). Measurement of the absorbance after solubilization of the formazan revealed higher metabolic activity in the sponge type scaffolds compared to the gel type scaffolds, in particular with PDGF. The absorbance was increased for the PDGF-loaded sponge type scaffolds (p<0.01). In addition, a time-course experiment was performed and formazan formation was assessed from day 1 to day 14. No increase of formazan formation over time in the groups was observed in the samples, which included the scaffolds and the “active zone” from day 1 to day 14 (Table S5).

### Gene expression dynamics

**Figure 6:**
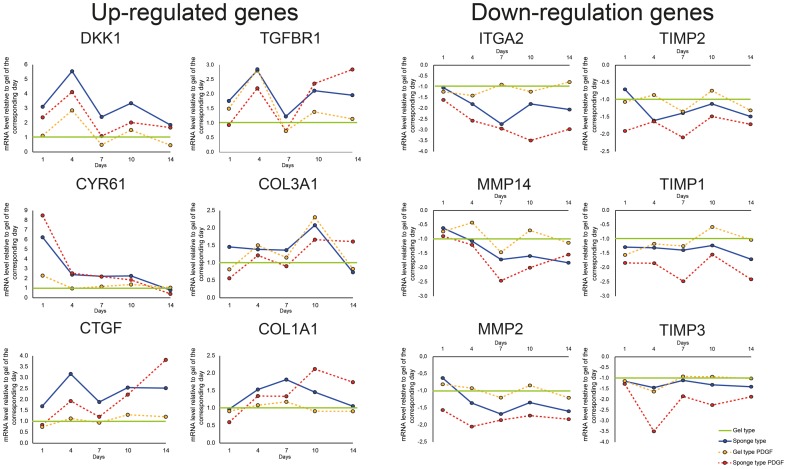
Gene expression profiles of DKK1, CYR61, CTGF, TGFBR1, COL3A1, COL1A1, ITGA2, MMP14, MMP2, TIMP2, TIMP1, TIMP3. The expression profiles of DKK1, CYR61, CTF, TGFBR, COL3A1, COL1A1 and ITGA2, MMP14, MMP2, TIMP2, TIMP1, TIMP3 up to 14 days in the gel type and sponge type scaffolds with and without platelet-derived growth factor-BB (PDGF) was measured using qPCR. (Up-regulated genes) Data shows x-fold increase of DKK1, CYR61, CTF, TGFBR, COL3A1, COL1A1 relative to gel type scaffold without PDGF at the corresponding time point (green line). (Down-regulated genes) Data shows x-fold decrease of ITGA2, MMP14, MMP2, TIMP2, TIMP1, TIMP3 relative to gel type scaffolds without PDGF at the corresponding time point (green line). Data points represent 5 samples

To reveal which genes are up-regulated and down-regulated by the scaffold in the early phase of population, we performed gene array analysis of the samples collected at day 1 (Table 1). To include the cells that are migrating towards the scaffold we isolated RNA from the sponge type scaffolds and the gel type scaffolds with the surrounding “active zone” as indicated (Figure 1A). We found that CTGF, CYR61, DKK1 were among the genes that were up-regulated 2-fold or more in the sponge type scaffolds compared to the gel type scaffolds. HAS2 and IGF2 were increased in the sponge type scaffolds with PDGF compared to the sponge type scaffolds. The gene array data was deposited into the National Center for Biotechnology Information Gene Expression Omnibus database with the accession numbers GSE61314, GSM1501968, GSM1501969, GSM1501970, and GSM1501971. Based on these results we proceeded to evaluate the gene regulation kinetics of the genes regulated in the scaffolds for up to 14 days using qPCR. In addition, we chose to analyze genes related to migration, proliferation, and matrix interaction (CCND1, ITGA2, ITGB1, PDGFRA, PDGFRB), matrix production (COLA1, COL3A1, FN1, POSTN, TNC), remodeling (MMP1, MMP2, MMP3, MMP14, TIMP1, TIMP2, TIMP3), and cell signaling factors (CCL2, IL-11). These genes were chosen based on their signal strength of above 1000, as determined by gene array, and their relevance in oral soft tissue. We found that the area under the curve above 1 for DKK1, CYR61, CTGF, TGFBR1, COL3A1, COL1A1 were increased (Table 1) over time indicating up-regulation. Further analysis revealed that CRY61, DKK1, and TGFBR1 showed an early response whereas COL1A1 and COL3A1 showed a later response in the model (Figure 6, see also Table S6 and S7 for the corresponding data). CRY61 expression peaked at day 1 whereas DKK1 and TGFBR1 peaked at day 4 in sponge type scaffolds with and without PDGF, and gel type scaffolds with PDGF relative to the gel type scaffolds. COL3A1 expression peaked at day 10 in sponge type scaffold, sponge type scaffold with PDGF, and gel type scaffolds with PDGF relative to the gel type scaffolds. In the sponge type scaffolds, CTGF peaked at day 4 whereas CTGF expression increased though day 14 in the sponge type scaffolds with PDGF. The CTGF expression in the gel type scaffolds with PDGF was not modulated. These results were consistent with the area under the curve analysis.

**Table 1:** Extract of gene expression.

Up-regulation				Down-regulation			
Area under the curve (day 1–14) from 2^−ddCt^>1				Area under the curve (day 1–14) from −1/2^−ddCt^<−1			
Gene Symbol	Sponge type	Sponge type PDGF	Gel type PDGF	Gene Symbol	Sponge type	Sponge type PDGF	Gel type PDGF
**DKK1**	**31.03**	16.70	6.60	**ITGA2**	**−12.55**	−24.07	−2.13
**CYR61**	**21.20**	24.20	3.92	**MMP14**	**−6.01**	−9.49	−0.73
**CTGF**	**18.67**	13.14	1.60	**MMP2**	**−5.24**	−10.79	−0.38
**TGFBR1**	**13.03**	11.25	7.28	*TIMP2*	−*4.78*	−9.65	−0.70
**COL3A1**	**6.73**	3.76	6.35	*TIMP1*	−*4.72*	−12.95	−1.84
**COL1A1**	**5.73**	7.19	0.64	*TIMP3*	−*3.75*	−16.57	−1.97
*POSTN*	*4.90*	17.99	14.84	*MMP1*	−*3.56*	−9.76	−0.33
*TNC*	*4.86*	4.39	5.24	*POSTN*	−*3.38*	n. d.	n. d.
*CCND1*	*4.62*	8.61	3.21	*PDGFRA*	−*3.17*	−9.74	−0.10
*HAS2*	*4.54*	36.24	16.40	*HAS2*	−*3.08*	n. d.	n. d.
*FN1*	*3.50*	0.65	0.78	*CCL2*	−*2.88*	−16.65	−1.44
*MMP3*	*3.18*	0.54	4.39	MMP3	−1.54	−32.94	−1.17
*ITGB1*	*2.32*	2.24	3.76	ITGB1	−0.25	−0.82	−0.46
*CCL2*	*2.03*	0.05	1.61	FN1	−0.13	−2.36	−1.63
MMP14	0.85	0.07	5.44	CCND1	−0.07	n. d.	−2.14
MMP1	0.77	0.62	2.09				
PDGFRA	0.14	0.03	2.49				

Fold changes in gene expression in the gel type and sponge type scaffolds including the “active zone” with and without platelet-derived growth factor-BB (PDGF) was assessed. The data is presented relative to the gel type scaffolds without PDGF. The area under the curve (AUC) above 1 and below −1 was calculated to indicate up-regulation and down-regulation, respectively. Genes are sorted based on the level of regulation in the scaffold. Up-regulation in the scaffold: **bold and underlined**>10; **bold**>5; *italic*>2; Down-regulation in the scaffold: **bold and underlined**<−10; **bold**<−5; *italic*<−2; n. d. = not determined.

Analysis of the area under the curve below −1 for ITGA2, MMP14, MMP2, TIMP1, TIMP2, and TIMP3 gene expression showed a decrease in expression over time (Table 1). Further analysis of the expression levels (Figure 6, see also Table S6 and S7 for the corresponding data) revealed that ITGA2 expression decreased in both sponge type scaffolds with and without PDGF with a maximum at day 10 and 7, respectively. For MMP14, MMP2, TIMP1, TIMP2, and TIMP3, a decrease of <−2-fold of gel was observed only in the sponge type scaffolds with PDGF. Overall, our results show that sponge type scaffolds increase DKK1, CYR61, CTGF, and TGFBR1 gene expression and decreases ITGA2 expression.

## Discussion

Graft consolidation follows a highly conserved sequence of wound healing, which includes activating platelets to release signaling molecules, such as platelet-derived growth factor isoforms, that then recruit gingival, mesenchymal repair cells [22]–[24]. These repair cells, including gingival fibroblasts, populate the wound healing site and facilitate the healing by the production of matrix and the release of signaling molecules in the defect [27], [28]. Here we provide evidence for cell population kinetics and gene expression dynamics involved in this reparative process using a novel *ex vivo* wound healing model with human gingival fibroblasts.

Early on in the model, fibroblasts begin to migrate from the adjacent matrix, the “active zone”, into the matrix of the scaffolds. These cells continued to populate the scaffolds over a period of 14 days. Addition of PDGF, mimicking the chemotactic effect of activated platelets, further increased the number of cells in the scaffolds and the mean distance from the scaffold border. This indicates that cells remain responsive to the chemotactic effects of PDGF in this model and is in line with the literature [23], [24]. This finding is supported by the enhanced levels of formazan formation in the scaffolds loaded with PDGF as determined by the MTT assay. Along with other studies, our model did not directly discriminate between PDGF induced proliferation and migration, as fibroblasts that undergo cell division remain DiI-positive [23], [24], [29]. We considered investigating the impact of proliferation on our model. The sponge type scaffold provides an environment that allows proliferation of human gingival fibroblasts seeded directly onto the scaffolds [25]. However, the increase in cell numbers over time is based on cells migrating from the active zone into the scaffolds, as the overall formation of formazan in the samples that include both, the scaffolds and the active zone, did not change over time (Table S2). This is further supported by an increase in the distance from the scaffold border over time and previous 2-D and 3-D *in vitro* studies [23], [24]. In our model, we used low serum concentrations, which, based on our MTT time course data and the literature do not support proliferation in 3-D cell cultures [30], [31]. These findings suggest that the differences between the gel type and sponge type scaffold group are due to migration and not proliferation. The difference between the PDGF-containing scaffolds and the scaffolds without PDGF can be caused by an initial increase in cell proliferation. However, time course experiments did not indicate an increase in proliferation from day 1 – day 14. This suggests that the increase in cell numbers within the scaffolds and distance of the cells from the scaffold border is based on migration. Indeed, there are strategies to further investigate the role of proliferation *in vitro* in addition to the well-established MTT assay [25], [32]. To prevent proliferation, anti-proliferative agents such as Mitomycin-C (MMC) are used in cell culture. However, under low serum conditions MMC has been shown to be cytotoxic [33], [34]. Therefore, under the conditions we used in our *ex vivo* wound-healing model, these cytotoxic effects can compromise the conclusions drawn from such experiments and is therefore not appropriate in this model. Taken together, our results indicate that the *ex vivo* model of oral wound healing presented here is a feasible way to determine cell population dynamics of collagen-based scaffolds.

Quantitative gene array analysis of the gingival fibroblasts, which had populated the scaffolds, revealed target genes associated with tissue repair. Further investigation of expression dynamics of these 25 genes from days 1 to 14 with quantitative PCR revealed that dickkopf WNT signaling pathway inhibitor 1 (DKK1), cysteine-rich, angiogenic inducer, 61 (CYR61), connective tissue growth factor (CTGF), transforming growth factor, beta receptor 1 (TGFBR1) were highly up-regulated and integrin α2 (ITGA2) was down-regulated in the sponge type scaffolds when compared to gel type scaffolds. Currently these genes have not been investigated for their role in scaffold supported gingival healing. The extra cellular matrix-related genes collagen type III α1 (COL3A1), collagen type I α1 (COL1A1), matrix metalloproteases (MMPs), and tissue inhibitor of metalloproteases (TIMPs) were moderately changed in scaffolds compared gels in our model.

DKK1 is an inhibitor of WNT signaling which is involved in cell proliferation and differentiation, particularly in the bone. DKK1 can also play a role in soft tissue healing as it can reduce proliferation of dermal fibroblasts [35], [36]. Thereby DKK1 can prevent fibrosis in skin regeneration [37]. However, DKK1 was also reported to increase skin thickness [38]. In our model, DKK1 is up-regulated in scaffolds when compared to gels. These initially high levels of DKK1 then decrease after day 4. Our finding that DKK1 levels are increased by sponge type scaffolds clearly indicates the importance of further research on the role of WNT signaling and DKK1 in gingival augmentation.

CYR61 and CTGF are two members of the CCN family that were both upregulated in the sponge type scaffolds compared to the gel type scaffolds. CYR61 is a matricellular protein with diverse functions [39], [40]. It can facilitate cell adhesion and migration and promote proliferation synergistically with growth factors. [39], [40]. CTGF has been shown to stimulate matrix production in gingival fibroblasts, which in turn contributes to gingival healing [41], [42]. Interestingly, CYR61 and CTGF show different expression dynamics in our system. CYR61 is highly expressed in the first days of culturing, which suggests that CYR61 facilitates the adhesion and migration of fibroblasts into the scaffold in the early phase. While CYR61 subsequently decreased, CTGF was stable in the sponge type scaffolds and increased with addition of PDGF. This finding suggests that these signaling molecules have distinct functions during wound healing.

TGFβ signaling is involved in fibroblast activation, proliferation, and matrix production. In our model, TGFBR1 gene expression was upregulated within hGFs in the sponge type scaffolds with and without PDGF, and in gel type scaffolds with PDGF at day 4 when compared to gel type scaffolds. This increase suggests that cells can become more susceptible to TGFβ in the early phase of sponge type scaffold population. However, TGFβ is involved in fibrosis and gingival overgrowth [36], [43]–[45]. TGFβ signaling also induces CTGF production [46]. CTGF can bind to TGFβ and increase its binding affinity to TGFBR1 [47]. Further studies will reveal the impact of sponge type scaffolds on this signaling pathway.

Among the genes that were downregulated in sponge type scaffolds compared to gel type scaffolds was the integrin ITGA2. ITGA2 is one of the integrins expressed during wound healing, and in combination with ITGB1 it facilitates cellular attachment to collagen and migration. Interestingly, the downregulation of ITGA2 was found in the sponge type scaffolds with and without PDGF containing group but not in the gel type scaffolds with PDGF, suggesting that the cells downregulate ITGA2 in the sponge type scaffolds. It is possible that cell attachment to the sponge type scaffolds involves a different integrin pattern from gel type scaffolds as their morphology and composition is different. Interestingly, other integrin isoforms that bind to collagen, such as ITGA1 and ITGB1, were not modulated by the sponge type scaffolds. As the number of fibroblasts in the gel type and the sponge type scaffolds increased in all groups, it is unlikely that the downregulation of ITGA2 in the sponge type scaffolds interfered with migration.

In the present study, we have shown that this *ex vivo* wound healing model can be used to analyze cellular population kinetics and gene expression dynamics of human primary gingival fibroblasts within experimental scaffolds. We used gel type scaffolds composed of rat tail collagen type I as a control to provide structural integrity of the *ex vivo* wound healing model and to ensure visibility of the defect margin. Cell-free BME alone did not allow for visualization of the border between cell-containing matrix and the defect over the observation period (data not shown). Gel type scaffolds composed of collagen type I, on the other hand, supported the structural integrity of the model and allowed visualization of the defect border. In addition, collagen type I is a well-established matrix for 3-D culture models that allows for migration of cells [23], [48]. It requires further investigation to determine the reasons for the differences between the gel type and the sponge type scaffold. Potential reasons are the architectural differences between the scaffolds. It is possible that migration into the gel type scaffolds requires more matrix remodeling than migration into the sponge type scaffold, which takes time and prolongs the process. Furthermore, the different biochemical composition may be the reason for the differences. While the gel is composed of rat-tail collagen type I, the sponge type scaffold is composed of porcine collagen types I and III [25].

In contrast to the gel type scaffolds, the sponge type scaffolds investigated herein are cross-linked, which can also contribute to the differences between the groups [12], [15], [18]. Although cross-linking collagen can interfere with biocompatibility *in vivo* and cellular activity *in vitro* [12], [15], [18], no toxic effects on the gingival fibroblasts were observed in our model. These results suggest that the cross-linking protocol has no negative impact on scaffold biocompatibility [19], [20]. This is in line with *in vitro* studies using bioreactors, which show proliferation of gingival fibroblasts in cross-linked, and in preclinical *in vivo* studies successfully grafting with the scaffold [25]. A limitation of our study is that our model does not consider mechanical forces that are applied during graft consolidation. However, in both *in vitro* and *in vivo* studies the sponge type scaffold composed of cross-linked collagen was shown to withstand these forces and provide volume support. *In vitro* fibroblasts responded to these forces with enhanced anabolic activity [19], [20], [25].

In addition to the function of providing volume support, the scaffold constructs can be used as carriers for recombinant growth factors such as PDGF, which provides mitogenic activity when healing is compromised. Preclinical animal studies using non cross-linked collagen membranes suggest that loading with growth factors is a feasible approach to accelerate soft tissue healing [49], [50]. In our study, loading with PDGF increased cell population and increased metabolic activity in the scaffolds indicating that the cells remain responsible to the growth factor. Scaffolds loaded with PDGF showed a stronger cellular response than collagen gel, suggesting that scaffolds with well-defined porous architecture have better carrier properties for PDGF. The herein presented *ex vivo* wound healing model will help to assess the impact on cellular population kinetics of future strategies to modify collagen scaffolds, such as loading with recombinant growth factors, gene vectors, and small molecules.

Furthermore, modification of the *ex vivo* wound healing model, including using other oral cells such as keratinocytes and endothelial cells, can shed light on other aspects of gingival regeneration. This includes insight into cell interactions and angiogenesis since cell behavior on scaffolds is modulated by the presence of other cell types [51], [52]. Including immune cells in this model will provide insight into the immune response to the scaffolds. What we have provided is a new tool by which we contribute to the understanding of the impact of biodegradable scaffolds on oral wound healing.

Overall, we have developed an *in vitro* 3-D model for analyzing scaffold population kinetics and gene expression of cells within collagen scaffolds. Our results show that the number of cells within the scaffold constructs and distance from the scaffold border increases over time in this new *ex vivo* model of oral wound healing repair. The absolute number of cells and distance was higher in the sponge type scaffolds when compared to gel type scaffolds. Loading the scaffolds with PDGF increased cell numbers, migration distance, and proliferation. Cells within sponge type scaffolds displayed increased expression of gene sets important for matrix biosynthesis including DKK1, CYR61, CTGF, TGFBR1 and decreased expression of integrin ITGA2. These genes may have an important role in oral soft tissue augmentation. This model system has strong potential as a tool for application in biomaterial oral wound healing kinetics. It will help to refine the materials and preclinical protocols, thereby contributing to the advancement of the field of oral soft tissue regeneration.

## Supporting Information

Table S1 **Gene expression assays.**
(PDF)

Table S2 **Corresponding data on cell numbers of human gingival fibroblasts in the gel type and the sponge type scaffolds.** Table S2 shows the corresponding data of Figure 3.
(XLSX)

Table S3 **Corresponding data on cell distance of human gingival fibroblasts in the gel type and sponge type scaffolds.** Table S3 shows the corresponding data of figure 4.
(XLSX)

Table S4 **Corresponding data on formazan formation of cells populating the gel type and sponge type scaffolds.** Table S4 shows the corresponding data of figure 5.
(XLSX)

Table S5 **Formazan formation of cells populating the gel type and sponge type scaffolds over time.** Formazan formation in the gel type and sponge type scaffolds with and without platelet-derived growth factor-BB (PDGF) including the “active zone” at day 1, 4, 7, 10, 14 was quantified utilizing the MTT assay. Data are given as mean ± standard deviation. (n = 6). Data was analyzed using analysis of variance (ANOVA) and Fisher's protected least significant difference (PLSD) *post hoc* test. Significance (*) vs. the corresponding day 1 was assigned at p<0.05.
(PDF)

Table S6 **Corresponding data on up-regulated genes.** Table S6 shows the corresponding data of the up-regulated genes in figure 6.
(XLSX)

Table S7 **Corresponding data on down-regulated genes.** Table S7 shows the corresponding data of the down-regulated genes in figure 6.
(XLSX)
