# Supplementary material for: Cell Population Kinetics of Collagen Scaffolds in Ex Vivo Oral Wound Repair
Source: PLoS One. 2014 Nov 14;9(11):e112680. doi: 10.1371/journal.pone.0112680 (PMC4232419; doi:10.1371/journal.pone.0112680)
Supplement: Table S1 — Gene expression assays. (PDF) [file pone.0112680.s001.pdf]

Table S1: Gene expression assays

Gene Symbol	Full name	Assays
CCL2	Chemokine (C-C motif) ligand 2	Hs00234140_m1
CCND1	Cyclin D1	Hs00765553_m1
COL1A1	Collagen, type I, alpha 1	Hs00164004_m1
COL3A1	Collagen, type III, alpha 1	Hs00943809_m1
CTGF	Connective tissue growth factor	Hs01026927_g1
CYR61	Cysteine-rich, angiogenic inducer, 61	Hs00998500_g1
DKK1	Dickkopf WNT signaling pathway inhibitor 1	Hs00183740_m1
FN1	Fibronectin 1	Hs00365052_m1
HAS2	Hyaluronan synthase 2	Hs00193435_m1
IGF2	Insulin-like growth factor 2 (somatomedin A)	Hs04188276_m1
IL-11	Interleukin 11	Hs01055413_g1
ITGA2	Integrin, alpha 2 (CD49B, alpha 2 subunit of VLA-2 receptor)	Hs00158127_m1
ITGB1	Integrin, beta 1 (fibronectin receptor, beta polypeptide, antigen CD29 includes MDF2, MSK12)	Hs00559595_m1
MMP1	Matrix metallopeptidase 1 (interstitial collagenase)	Hs00899658_m1
MMP14	Matrix metallopeptidase 14 (membrane-inserted)	Hs01037009_g1
MMP2	Matrix metallopeptidase 2 (gelatinase A, 72kDa gelatinase, 72kDa type IV collagenase)	Hs01548727_m1
MMP3	matrix metallopeptidase 3 (stromelysin 1, progelatinase)	Hs00968305_m1
PDGFRA	Platelet-derived growth factor receptor, alpha polypeptide	Hs00998018_m1
PDGFRB	Platelet-derived growth factor receptor, beta polypeptide	Hs01019589_m1
POSTN	Periostin, osteoblast specific factor	Hs01566734_m1
TGFBR1	Transforming growth factor, beta receptor 1	Hs00610320_m1
TIMP1	TIMP metallopeptidase inhibitor 1	Hs00171558_m1
TIMP2	TIMP metallopeptidase inhibitor 2	Hs00234278_m1
TIMP3	TIMP metallopeptidase inhibitor 3	Hs00165949_m1
TNC	Tensascin c	Hs01115665_m1
