# Supplementary material for: Cell Population Kinetics of Collagen Scaffolds in Ex Vivo Oral Wound Repair
Source: PLoS One. 2014 Nov 14;9(11):e112680. doi: 10.1371/journal.pone.0112680 (PMC4232419; doi:10.1371/journal.pone.0112680)
Supplement: Table S5 — Formazan formation of cells populating the gel type and sponge type scaffolds over time. Formazan formation in the gel type and sponge type scaffolds with and without platelet-derived growth factor-BB (PDGF) including the “active zone” at day 1, 4, 7, 10, 14 was quantified utilizing the MTT assay. Data are given as mean ± standard deviation. (n = 6). Data was analyzed using analysis of variance (ANOVA) and Fisher's protected least significant difference (PLSD) post hoc test. Significance (*) vs. the corresponding day 1 was assigned at p<0.05. (PDF) [file pone.0112680.s005.pdf]

Table S5: Formazan formation of cells populating the gel type and sponge type scaffolds over time.

	**Day 1**	**Day 4**	**Day 7**	**Day 10**	**Day 14**
**Sponge type scaffold**	0.204±0.023	0.177±0.019*	0.193±0.027	0.177±0.020*	0.210±0.023
**Sponge type scaffold PDGF**	0.254±0.039	0.210±0.032*	0.252±0.028	0.246±0.033	0.245±0.049
**Gel type scaffold**	0.170±0.021	0.139±0.018*	0.148±0.015	0.134±0.015*	0.150±0.044
**Gel type scaffold PDGF**	0.170±0.028	0.185±0.020	0.178±0.014	0.143±0.017*	0.157±0.046

Formazan formation in the gel type and sponge type scaffolds with and without platelet-derived growth factor-BB (PDGF) including the “active zone” at day 1, 4, 7, 10, 14 was quantified utilizing the MTT assay. Data are given as mean ± standard deviation. (n=6). Data was analyzed using analysis of variance (ANOVA) and Fisher’s protected least significant difference (PLSD) *post hoc* test. Significance (*) vs. the corresponding day 1 was assigned at $p<0.05$.
